# Comparative Efficacy and Cycle-Wise Toxicity of Paclitaxel-Carboplatin Versus Gemcitabine-Cisplatin in Stage IV Non-small Cell Lung Cancer (NSCLC): A Quasi-experimental Study in Bangladesh

**DOI:** 10.7759/cureus.93093

**Published:** 2025-09-24

**Authors:** Md. Raihan Bin Sharif, A.M.M. Shariful Alam, H.M. Nayeem Iqbal, Salman Bashar A Ayub, Mohammad Habibur Rahaman

**Affiliations:** 1 Department of Medical Oncology, Ahsania Mission Cancer and General Hospital, Dhaka, BGD; 2 Department of Clinical Oncology, Ahsania Mission Cancer and General Hospital, Dhaka, BGD; 3 Department of Radiotherapy, Dhaka Medical College Hospital, Dhaka, BGD; 4 Department of Oncology, National Institute of Cancer Research and Hospital, Dhaka, BGD

**Keywords:** chemotherapy toxicity, gemcitabine–cisplatin, nsclc, paclitaxel–carboplatin, platinum-based doublet therapy

## Abstract

Background

Non-small cell lung cancer (NSCLC) remains a leading cause of cancer mortality worldwide. In Bangladesh, limited access to novel therapeutics makes platinum-based chemotherapy the mainstay of treatment. This study compares the efficacy and cycle-wise toxicity profiles of paclitaxel-carboplatin versus gemcitabine-cisplatin in Stage IV NSCLC patients.

Methods

In this quasi-experimental study, 66 Stage IV NSCLC patients at Ahsania Mission Cancer & General Hospital, Dhaka, received paclitaxel-carboplatin (Group A) or gemcitabine-cisplatin (Group B). Tumor response, six- and nine-month survival, and hematologic (e.g., neutropenia, thrombocytopenia) and non-hematologic (e.g., nausea, vomiting) toxicities were assessed over three chemotherapy cycles. Statistical analysis included Chi-square, Mann-Whitney, or t-tests; p < 0.05 was considered significant.

Results

A partial response was observed in 18 (54.6%) of patients in both groups, with no complete responses recorded. Disease control rates were 26 (78.8%) in Group A and 25 (75.8%) in Group B (p = 0.716). Six-month survival rates were 18 (54.5%) and 20 (60.9%). Nine-month survival rates decreased to 10 (30.3%) and 12 (36.4%), respectively. Group A experienced significantly higher rates of leukopenia and neutropenia across all cycles, with six-cycle incidences of 25 (75.8%) and 26 (78.8%), respectively (p < 0.05). At the same time, thrombocytopenia was notably more common in Group B, with 21 (63.6%) over six cycles (p < 0.05). Non-hematologic toxicities were mild and statistically similar between groups.

Conclusions

Paclitaxel-carboplatin and gemcitabine-cisplatin demonstrated comparable efficacy and short-term survival. However, their distinct hematologic toxicity profiles highlight the need for individualized regimen selection based on patient tolerance and supportive capacity in resource-limited settings.

## Introduction

Lung cancer remains the leading cause of cancer-related mortality globally, with non-small cell lung cancer (NSCLC) accounting for approximately 80-85% of all lung cancer cases [1, 2]. In 2020 alone, an estimated 2.2 million new cases of lung cancer were diagnosed worldwide, resulting in nearly 1.8 million deaths [3]. The majority of patients are diagnosed at an advanced stage (Stage III or IV), where curative options are limited, and systemic therapy becomes the cornerstone of management [1]. In low- and middle-income countries (LMICs) such as Bangladesh, the burden of NSCLC is rising rapidly due to increasing tobacco consumption, industrial pollution, and limited access to early diagnostic facilities [4, 5].

For patients with Stage IV NSCLC who lack actionable driver mutations and are ineligible for immunotherapy, platinum-based doublet chemotherapy remains the recommended first-line treatment according to ESMO Clinical Practice Guidelines [6]. Among the cytotoxic options for advanced NSCLC, commonly used platinum-based doublets include paclitaxel-carboplatin and gemcitabine-cisplatin. Randomized trials, including a four-arm study published in the *New England Journal of Medicine *(*NEJM*), have demonstrated comparable overall survival rates across these standard doublets [7, 8]. However, toxicity profiles, ease of administration, patient tolerability, and institutional capacity often determine regimen selection in real-world settings.

Paclitaxel-carboplatin is known for its relatively favorable nephrotoxicity profile and shorter infusion times, but carries a higher risk of hematologic toxicity, particularly neutropenia and peripheral neuropathy [9]. Conversely, gemcitabine-cisplatin has demonstrated lower neurotoxicity but is associated with more frequent renal and hematologic adverse effects, including thrombocytopenia and nausea [10, 11]. These variations in toxicity profiles are particularly relevant in LMICs, where supportive care resources such as granulocyte colony-stimulating factors and nephroprotective agents may be limited. Furthermore, differences in drug cost, availability, and scheduling requirements play a critical role in regimen feasibility and compliance in under-resourced oncology centers [12, 13].

Despite the widespread use of both regimens globally, there is a paucity of localized comparative data evaluating their real-world effectiveness and tolerability in South Asian populations, where evidence gaps in lung cancer treatment have been well documented [14]. Most clinical trials originate from high-income countries and may not fully reflect the sociodemographic and healthcare realities of LMICs like Bangladesh, where patient characteristics, comorbidity profiles, and treatment infrastructure differ significantly [13]. Additionally, detailed cycle-wise data on toxicity progression and treatment response are seldom reported in the existing literature [15]. However, such insights are vital for guiding clinicians in making decisions about dose modification and treatment continuation in standard clinical practice.

Recognizing gaps in locally relevant evidence, we conducted a quasi-experimental study to systematically compare the efficacy and cycle-wise toxicity profiles of paclitaxel-carboplatin versus gemcitabine-cisplatin in patients with Stage IV NSCLC at a tertiary cancer center in Bangladesh. The study aimed to compare tumor response, cycle-wise hematologic and non-hematologic toxicity profiles, and short-term survival outcomes of paclitaxel-carboplatin versus gemcitabine-cisplatin in patients with Stage IV NSCLC, tailored to patient tolerability and resource availability in a low-resource oncology setting.

## Materials and methods

Study design and setting

This study employed a prospective, quasi-experimental design, which is suitable for a real-world clinical setting where randomization was not feasible. Patients were assigned to treatment groups based on the treating oncologist's clinical judgment and patient preference, reflecting routine clinical decision-making, and this approach increases external validity and real-world applicability.

The study was conducted at the Department of Medical Oncology, National Institute of Cancer Research and Hospital (NICRH), Dhaka, Bangladesh, over eighteen months, from January 2022 to August 2023. The research protocol was developed in advance and received ethical approval from the Institutional Review Board of the National Institute of Cancer Research and Hospital (NICRH), Dhaka (Approval No. NICHR/IRB/2022/305). All participants provided written informed consent before enrolment.

Study participants

Patients were enrolled consecutively based on predefined eligibility criteria. Eligible participants were adults aged 18 to 80 years with a histologically or cytologically confirmed diagnosis of Stage IV non-small cell lung cancer (NSCLC), a performance status of 0 to 2 on the World Health Organization (WHO)/Eastern Cooperative Oncology Group (ECOG) scale, and bidimensional measurable disease. Additional inclusion criteria required adequate hematologic function, defined as a white blood cell count ≥4,000/mm³, platelet count ≥100,000/mm³, and hemoglobin ≥10 g/dL. Female patients of childbearing potential were required to use effective contraception.

Patients were excluded if they had previously received chemotherapy, immunotherapy, or radiotherapy; had significant comorbidities or organ dysfunction (e.g., renal or hepatic impairment) contraindicating platinum-based chemotherapy; or were unable to complete the planned chemotherapy cycles due to poor performance status or voluntary withdrawal.

**Figure 1:**
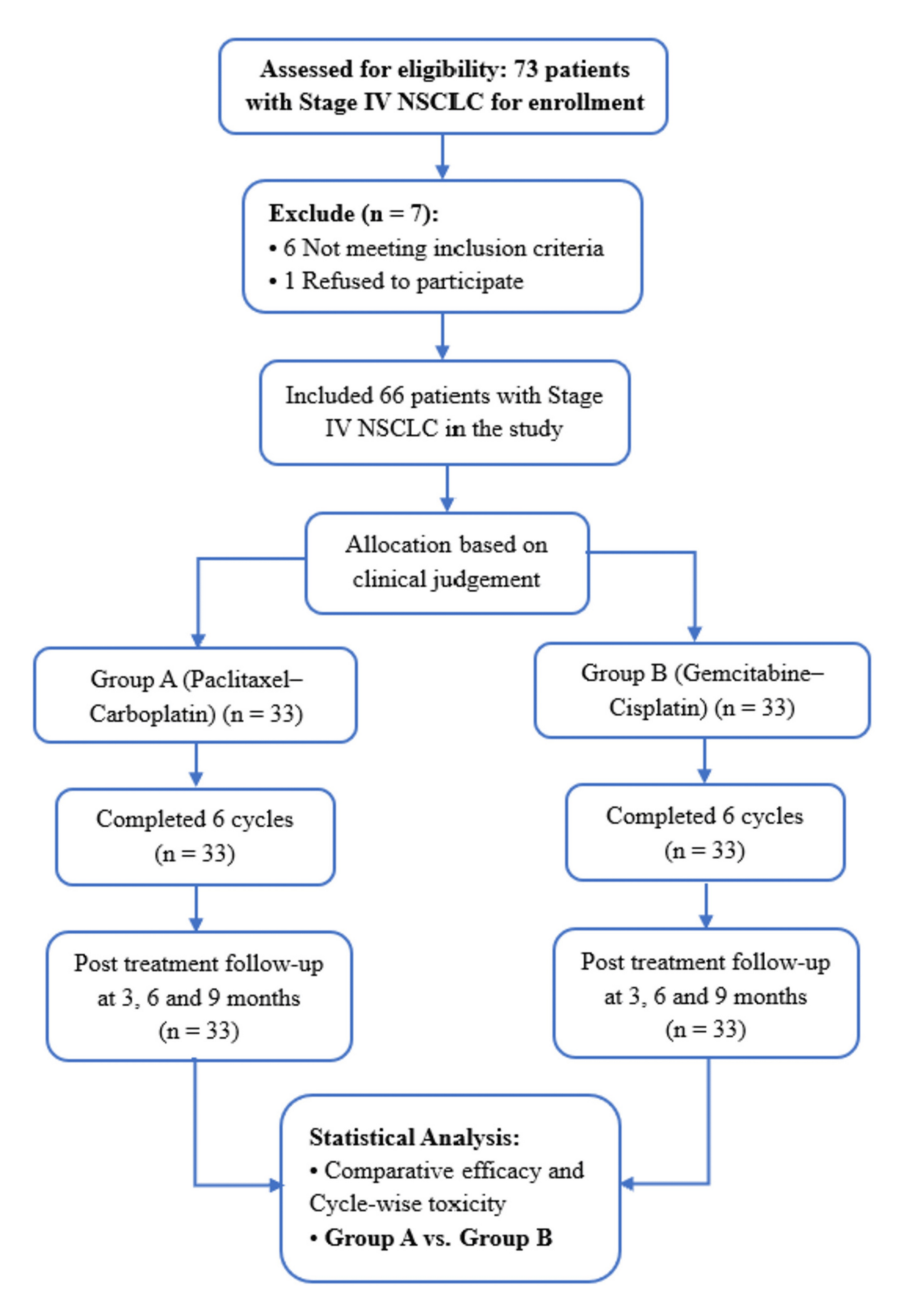
Flowchart of patient screening, enrollment, allocation, and follow-up. A total of 73 patients were screened, with seven excluded (six ineligible, one declined participation). Sixty-six patients were enrolled and equally assigned to Group A (paclitaxel–carboplatin) or Group B (gemcitabine–cisplatin) and followed for treatment outcomes and survival at six and nine months. NSCLC: non-small cell lung cancer

During the study period, a total of 73 patients were assessed for eligibility. After applying predefined inclusion and exclusion criteria, 66 patients were enrolled and equally allocated into two treatment groups (n = 33 per group). This sample size was adequate based on the initial sample size calculation. Treatment allocation was non-randomized and based on physician judgment and patient preference, reflecting routine clinical practice in this setting. This approach mirrors real-world decision-making. The study participant flowchart is shown in Figure 1.

Sample size calculation

A purposive sampling method was used to recruit eligible patients into two treatment groups consecutively. The sample size was calculated based on the anticipated difference in the proportion of leukopenia between two platinum-based chemotherapy regimens: gemcitabine-cisplatin and paclitaxel-carboplatin, both of which are commonly employed in the treatment of Stage IV non-small cell lung cancer (NSCLC). The following formula was utilized for sample size estimation:

$n = \frac{P_1(1-P_1)+P_2(1-P_2)}{(P_1-P_2)^2}&times;(Z_\alpha+Z_\beta )^2$

where P₁ = 93.33% (gemcitabine-cisplatin group), P₂ = 63.33% (paclitaxel-carboplatin group), Z_α_ = 1.96 (95% confidence), and Z_β_ = 1.28 (90% power). The calculated sample size was approximately 32.71 per group. These proportions were derived from leukopenia rates reported by Wang et al. (2022) [16]. Rounding up, 33 patients were enrolled in each group, resulting in a total sample size of 66.

Pretreatment evaluation

All patients underwent a comprehensive pretreatment evaluation, including detailed medical history, physical examination, and performance status assessment. Baseline investigations included complete blood count, urinalysis, serum biochemistry, and electrocardiogram (ECG). Radiologic assessments included chest X-ray, ultrasonography (whole abdomen), whole-body bone scan, brain computed tomography (CT), and chest CT with liver and adrenal gland visualization. Laboratory tests were completed within 7 days prior to study entry, and imaging assessments were performed within 14 days before enrollment.

Intervention

Eligible patients were assigned to one of two treatment arms based on the attending oncologist’s recommendation and patient preference, without randomization. Each group received a distinct platinum-based doublet chemotherapy regimen administered every three weeks, constituting one treatment cycle. Treatment was administered every three weeks for up to six cycles, unless discontinued due to progression or adverse events.

Group A (paclitaxel-carboplatin regimen)

Patients in this arm received paclitaxel at a dose of 200 mg/m² administered as a three-hour intravenous infusion on Day 1, followed by carboplatin calculated to an area under the curve (AUC) of 6 using the Calvert formula, administered as a one-hour intravenous infusion. Paclitaxel administration was preceded by standard premedication to minimize hypersensitivity reactions, including dexamethasone 20 mg orally 12 hours before and intravenously one hour before infusion, diphenhydramine 50 mg intravenously 30 minutes before paclitaxel, and ondansetron 16 mg intravenously 15 minutes before chemotherapy, followed by 8 mg orally twice daily for the next three days.

Group B (gemcitabine-cisplatin regimen)

Patients assigned to this group received gemcitabine 1250 mg/m² intravenously on Days 1 and 8, in combination with cisplatin 75 mg/m² administered intravenously on Day 1. This regimen was also repeated every three weeks. One cycle was considered complete upon administration of both gemcitabine doses per cycle.

Patients in both groups were monitored clinically and radiologically for disease status at scheduled intervals. If a partial response or disease stability was observed, treatment continuation was determined at the physician’s discretion. Chemotherapy was withheld in cases of disease progression or unacceptable adverse events.

Outcome measures

Primary Efficacy Assessment

Tumor response was evaluated according to the Response Evaluation Criteria in Solid Tumors (RECIST), version 1.1 [17, 18]. Radiologic assessments, including contrast-enhanced computed tomography (CT) of the chest and upper abdomen, were performed at mid-treatment (after the third cycle) and at the end of treatment (after the sixth cycle). Interim chest X-rays were conducted every three weeks to monitor interval changes. Histopathological diagnoses were confirmed by a consistent panel of pathologists to ensure interpretive reliability.

Under RECIST v1.1, a complete response was defined as the disappearance of all measurable target lesions. Partial response was assigned when there was at least a 30% decrease in the sum of the diameters of target lesions compared to baseline. Progressive disease was defined as a minimum 20% increase in the sum of diameters or the appearance of new lesions. Stable disease refers to changes that are insufficient to qualify as either partial response or progression. The disease control rate (DCR) was calculated as the proportion of patients achieving complete response, partial response, or stable disease. Patients who completed at least two cycles of therapy were considered evaluable for tumor response. Time to progression (TTP) was measured from chemotherapy initiation to documented disease progression, and overall survival (OS) from chemotherapy initiation to death from any cause.

Secondary Endpoint (Toxicity) Assessment

Adverse events were recorded at the end of each treatment cycle, allowing for cycle-wise analysis. Toxicity profiles were assessed separately for each chemotherapy cycle (cycle-wise) to evaluate how adverse effects evolved over successive treatment cycles of paclitaxel-carboplatin versus gemcitabine-cisplatin in Stage IV NSCLC patients. Treatment-related adverse events were graded according to the Common Terminology Criteria for Adverse Events (CTCAE), version 5.0 [19], which classifies severity on a five-grade scale ranging from Grade 1 (mild) to Grade 5 (death). Toxicities were evaluated after the first, third, and sixth cycles of chemotherapy. Both hematologic (e.g., leukopenia, neutropenia, thrombocytopenia) and non-hematologic (e.g., nausea, vomiting, hepatotoxicity, renal impairment) adverse events were recorded, with special attention to Grade 3 and Grade 4 events, which were deemed clinically significant.

Treatment Modifications

Patients in Group A received paclitaxel 200 mg/m² plus carboplatin AUC 6, while those in Group B received gemcitabine 1250 mg/m² plus cisplatin 75 mg/m², all at full doses. Dose reductions were pre-specified for patients experiencing hematologic or severe non-hematologic toxicities, following a four-level sequence: Level 1 (initial full dose), Level 2 (first reduction), Level 3 (second reduction), and Level 4 (third reduction). Once doses were reduced, they were not re-escalated. Hematologic recovery was monitored through twice-weekly complete blood counts (CBCs). Patients were to be withdrawn if recovery was not achieved by day 35 or if severe cardiac, hepatic, or neurologic toxicities occurred. These measures were implemented to ensure patient safety in accordance with standard oncology protocols.

Statistical analysis

All data were entered into and analyzed using IBM SPSS Statistics for Windows, Version 25.0 (IBM Corp., Armonk, NY, USA). Categorical variables such as response rates and adverse events were analyzed using the Chi-square test or Fisher's exact test, as appropriate. For toxicity grading, the five-grade CTCAE scale (Grade 1 = mild to Grade 5 = death) was dichotomized into clinically significant (Grades 3-4) versus lower-grade (Grade 1-2) events for comparative analysis. Continuous variables such as age were expressed as mean ± standard deviation and analyzed using an independent samples t-test. Because the toxicity and hemoglobin count data were not normally distributed, they were summarized as median (interquartile range, IQR) and analyzed using the Mann-Whitney test for between-group comparisons. A p-value of less than 0.05 was considered statistically significant.

## Results

Baseline demographic and clinical characteristics

The age distribution among patients was similar across both groups. In Group A (Paclitaxel-Carboplatin), eight (24.2%) patients were aged 40-50 years, 22 (66.7%) were 50-60 years, and three (9.1%) were over 60 years old. In Group B (Gemcitabine-Cisplatin), seven (21.2%) of the patients were aged 40-50 years, 21 (63.6%) were 50-60 years, and five (15.2%) were over 60 years old. The mean age was 54.3 ± 8.3 years in Group A and 54.4 ± 12.3 years in Group B (p = 0.972).

Male patients accounted for 21 (63.6%) in Group A and 23 (69.7%) in Group B (p = 0.794). A history of smoking was reported in 24 (72.7%) of patients in Group A and 28 (84.8%) in Group B (p = 0.367). ECOG performance status of 0-1 was observed in 26 (78.8%) of patients in Group A and 25 (75.8%) in Group B (p = 0.774).

Regarding histological subtype, squamous cell carcinoma was diagnosed in 12 (36.4%) of patients in Group A and 10 (30.3%) in Group B. Adenocarcinoma was more common, found in 21 (63.6%) of patients in Group A and 23 (69.7%) in Group B (p = 0.794)

Baseline hematological parameters were comparable between the two groups. The median hemoglobin level was 12.0 g/dL (IQR: 1.3) in Group A and 12.0 g/dL (IQR: 0.9) in Group B, showing no statistically significant difference (p = 0.919). The mean baseline white blood cell count was 5963.6 ± 1245.4 ×10³/mm³ in Group A and 5569.7 ± 1311.8 ×10³/mm³ in Group B (p = 0.107). Similarly, the mean platelet counts were 175130.3 ± 13896.1 ×10³/mm³ in Group A and 173584.8 ± 14996.6 ×10³/mm³ in Group B, with no significant difference (p = 0.332) (Table 1).

**Table 1:** Baseline demographic and clinical characteristics of study participants (n = 66) Group A: paclitaxel–Carboplatin, Group B: gemcitabine–cisplatin, ECOG: Eastern Cooperative Oncology Group. Values are presented as numbers (percentages), p-values obtained using Chi-square tests, and Fisher’s exact test (for small values) for categorical variables and independent t-tests for continuous variables. Note: Smoking history was defined as current or former smoking for ≥6 consecutive months before diagnosis.

Variable	Group A (n = 33; %)	Group B (n = 33; %)	p-value
Age Group (years)			
40–50	8 (24.2)	7 (21.2)	0.869
50–60	22 (66.7)	21 (63.6)	
>60	3 (9.1)	5 (15.2)	
Age (years), Mean ± SD	54.3 ± 8.3	54.4 ± 12.3	0.972
Sex (Male)	21 (63.6)	23 (69.7)	0.794
Smoking History	24 (72.7)	28 (84.8)	0.367
ECOG Performance (0–1)	26 (78.8)	25 (75.8)	0.774
Histology			
Squamous cell carcinoma	12 (36.4)	10 (30.3)	0.794
Adenocarcinoma	21 (63.6)	23 (69.7)	
Hematological Values			
Hemoglobin (g/dL), Median (IQR)	12 (1.3)	12 (0.9)	0.919
White Blood Cell Count (×10³/mm³), Mean ± SD	5963.6 ± 1245.4	5569.7 ± 1311.8	0.107
Platelet Count (×10^3^/mm^3^), Mean ± SD	175130.3 ± 13896.1	173584.8 ± 14996.6	0.332

Treatment response profile of paclitaxel-carboplatin and gemcitabine-cisplatin regimens in stage IV NSCLC patients

Among the 66 patients evaluated for treatment response, no cases of complete response (CR) were observed in either treatment group. A partial response (PR) was noted in 18 (54.6%) of patients in both Group A (Paclitaxel-Carboplatin) and Group B (Gemcitabine-Cisplatin). Stable disease (SD) was observed in eight (24.2%) in Group A, and in seven (21.2%) in Group B. Progressive disease (PD) occurred in seven (21.2%) in Group A, and in eight (24.2%) in Group B. The overall disease control rate (CR + PR + SD) was 26 (78.8%) in Group A and 25 (75.8%) in Group B. The difference in response distribution between the two groups was not statistically significant (p = 0.716) (Table 2).

**Table 2:** Distribution of treatment response among NSCLC patients (n = 66) Group A: paclitaxel–Carboplatin, Group B: gemcitabine–cisplatin. Responses were evaluated per Response Evaluation Criteria in Solid Tumors (RECIST) criteria, DCR = CR + PR + SD. Values are presented as numbers (percentages). p-value obtained using the Chi-square test, for the overall distribution of treatment response between groups. NSCLC: non-small cell lung cancer

Treatment Response	Group A (n = 33; %)	Group B (n = 33; %)	p-value
Complete Response (CR)	0 (0.0)	0 (0.0)	0.716
Partial Response (PR)	18 (54.6)	18 (54.6)	
Stable Disease (SD)	8 (24.2)	7 (21.2)	
Progressive Disease (PD)	7 (21.2)	8 (24.2)	
Disease Control Rate (CR + PR + SD)	26 (78.8)	25 (75.8)	

Short-term survival outcomes

At six months post-treatment, 18 (54.5%) of patients in the paclitaxel-carboplatin group and 20 (60.9%) in the gemcitabine-cisplatin group were alive (χ² = 0.2481, df = 1, p = 0.618). At nine months, survival decreased to 10 (30.3%) of patients in Group A and 12 (36.4%) in Group B (χ² = 0.2727, df = 1, p = 0.602). Mortality rates were correspondingly higher at each time point, but no statistically significant differences were observed between the two groups at either follow-up interval (Table 3).

**Table 3:** Survival outcomes at six- and nine-months post-treatment (n = 66) Group A: paclitaxel–carboplatin, Group B: gemcitabine–cisplatin. Survival status was assessed at two follow-up points: six months and nine months after the initiation of chemotherapy. Values are presented as numbers (percentages). p-values were calculated using the Chi-square test.

Post-treatment duration	Survival status	Group A (n = 33; %)	Group B (n = 33; %)	p-value
6 months	Survived	18 (54.5)	20 (60.9)	0.618
	Died	15 (45.5)	13 (39.1)	
9 months	Survived	10 (30.3)	12 (36.4)	0.602
	Died	23 (69.7)	21 (63.6)	

Hematologic toxicities

During the first chemotherapy cycle, grade ≥2 leukopenia was reported in 29 (87.9%) in the Paclitaxel-Carboplatin group (Group A), significantly higher than the 13 (39.4%) in the Gemcitabine-Cisplatin group (Group B) (p < 0.001). In the third cycle, leukopenia remained more frequent in Group A, affecting 27 (81.8%) of patients, compared to 13 (39.4%) in Group B (p < 0.001). By the sixth cycle, leukopenia was observed in 25 (75.8%) in Group A and 11 (33.3%) in Group B (p = 0.001).

Grade ≥2 neutropenia was more common in Group A, affecting 28 (81.8%) of patients during the first cycle, compared to 18 (54.5%) in Group B (p = 0.015). This trend continued in the third and sixth cycles, where 26 (78.8%) of patients in Group A experienced neutropenia, significantly higher than the 17 (51.5%) in Group B (p = 0.038 for both cycles).

In contrast, grade ≥2 thrombocytopenia was more prevalent in Group B across all cycles. During the first cycle, 23 (69.7%) of patients in Group B experienced thrombocytopenia, compared to 13 (39.4%) in Group A (p = 0.013). In the third cycle, 23 (69.7%) of patients in Group B were significantly affected, compared to 11 (33.3%) in Group A (p = 0.026). By the sixth cycle, thrombocytopenia was reported in 21 (63.6%) in Group B, more common than in 12 (36.4%) in Group A (p = 0.021) (Table 4).

**Table 4:** Cycle-wise hematologic toxicities (Grade ≥2) based on the CTCAE (version 5.0) (n = 66) Group A: paclitaxel–carboplatin, Group B: gemcitabine–cisplatin, CTCAE: Common Terminology Criteria for Adverse Events. Values are presented as numbers (percentages), p-values obtained using the Chi-square test, *statistically significant at p < 0.05

Toxicity Type	Cycle	Group A (n = 33; %)	Group B (n = 33; %)	p-value
Leukopenia	1st	29 (87.9)	13 (39.4)	<0.001*
	3rd	27 (81.8)	13 (39.4)	<0.001*
	6th	25 (75.8)	11 (33.3)	<0.001*
Neutropenia	1st	28 (81.8)	18 (54.5)	0.015*
	3rd	26 (78.8)	17 (51.5)	0.038*
	6th	26 (78.8)	17 (51.5)	0.038*
Thrombocytopenia	1st	13 (39.4)	23 (69.7)	0.013*
	3rd	11 (33.3)	23 (69.7)	0.026*
	6th	12 (36.4)	21 (63.6)	0.021*

Non-hematologic toxicities

In the first chemotherapy cycle, nausea was reported in 29 (87.9%) of patients in the paclitaxel-carboplatin group (Group A) and 24 (72.8%) in the gemcitabine-cisplatin group (Group B) (p = 0.215). Similar patterns were observed in the third cycle, with 28 (84.8%) in Group A and 23 (69.7%) in Group B reporting nausea (p = 0.240), and in the sixth cycle, where 21 (63.6%) in Group A and 18 (54.5%) in Group B experienced nausea (p = 0.617).

Vomiting was documented in seven (21.2%) patients in Group A and nine (27.3%) in Group B during the first cycle (p = 0.775). In the third cycle, six (18.2%) patients in Group A and eight (24.2%) in Group B reported vomiting (p = 0.764). By the sixth cycle, vomiting was noted in six (18.2%) in Group A and five (15.2%) in Group B (p = 1.000).

Elevated serum glutamic pyruvic transaminase (SGPT) levels were observed in two (6.1%) patients in Group A and four (12.1%) in Group B during the first cycle (p = 0.803). In the third cycle, four (12.1%) of patients in Group A and two (6.1%) in Group B had elevated SGPT (p = 0.803); this trend persisted in the sixth cycle with the same distribution (p = 0.803).

Elevated serum creatinine was reported in two (6.1%) of patients in Group A and three (9.1%) in Group B during the first cycle (p = 1.000). In the third cycle, elevated creatinine was found in two (6.1%) of patients in Group A and four (12.1%) in Group B (p = 0.803), with similar findings continuing in the sixth cycle (p = 1.000) (Table 5).

**Table 5:** Cycle-wise non-hematologic toxicities (all grades) based on the CTCAE (version 5.0) (n = 66) Group A: paclitaxel–carboplatin, Group B: gemcitabine–cisplatin, CTCAE: Common Terminology Criteria for Adverse Events, SGPT: Serum Glutamate Pyruvate Transaminase. Values are presented as numbers (percentages), p-values obtained using the Chi-square test, and Fisher’s exact test (for small values)

Toxicity Type	Cycle	Group A (n=33; %)	Group B (n=33; %)	p-value
Nausea	1st	29 (87.9)	24 (72.8)	0.215
	3rd	28 (84.8)	23 (69.7)	0.240
	6th	21 (63.6)	18 (54.5)	0.617
Vomiting	1st	7 (21.2)	9 (27.3)	0.775
	3rd	6 (18.2)	8 (24.2)	0.764
	6th	6 (18.2)	5 (15.2)	1.000
Elevated SGPT	1st	2 (6.1)	4 (12.1)	0.803
	3rd	4 (12.1)	2 (6.1)	0.803
	6th	4 (12.1)	2 (6.1)	0.803
Elevated Creatinine	1st	2 (6.1)	3 (9.1)	1.000
	3rd	2 (6.1)	4 (12.1)	0.803
	6th	2 (6.1)	3 (9.1)	1.000

Median adverse event profiles across chemotherapy cycles

**Figure 2:**
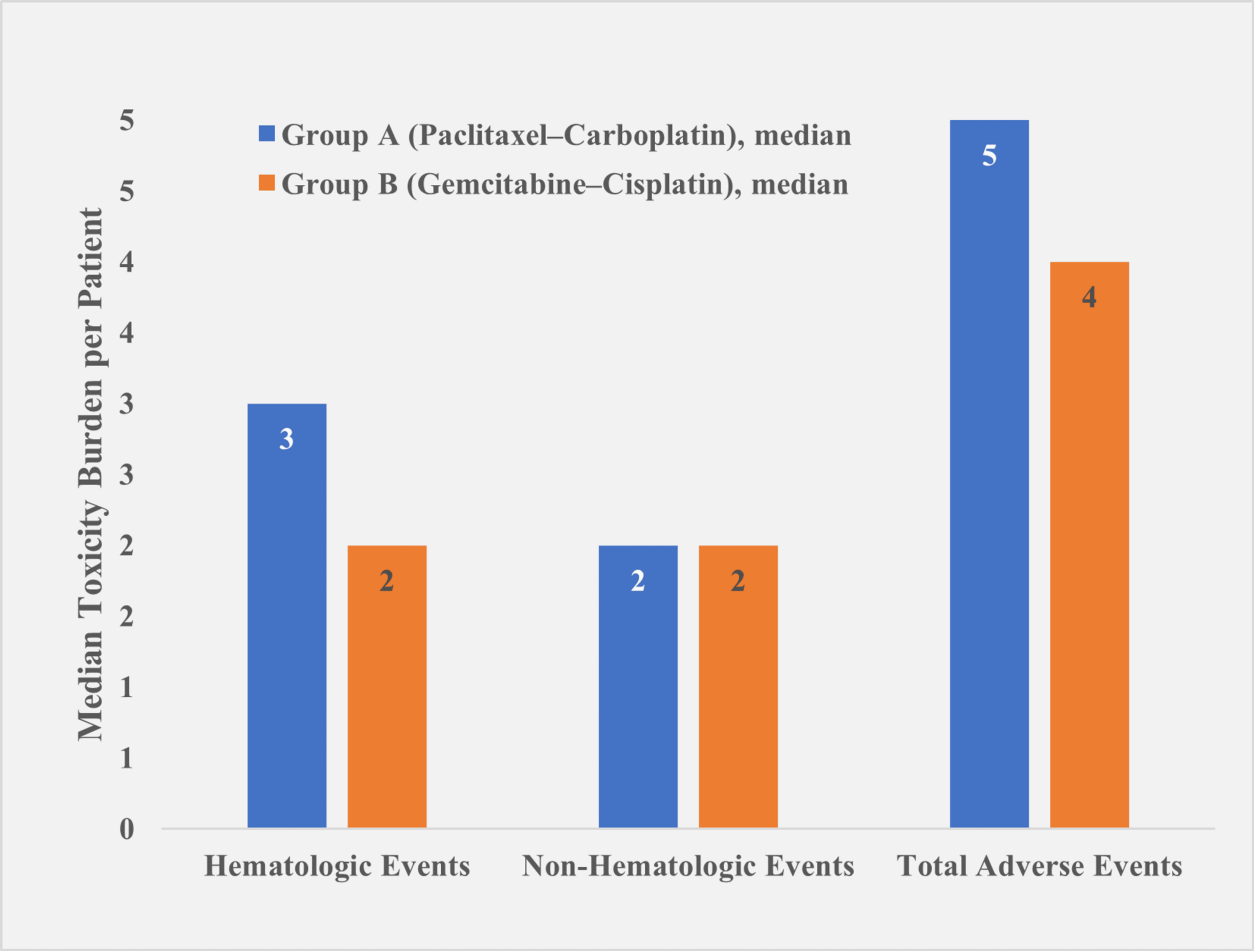
Median toxicity burden per patient across three chemotherapy cycles (n = 66) Group A: paclitaxel–carboplatin, Group B: gemcitabine–cisplatin. Group A demonstrated a higher median hematologic toxicity burden per patient compared to Group B across three chemotherapy cycles (3.0, IQR 2.0–3.0 vs. 2.0, IQR 1.0–2.0; p = 0.029). Median non-hematologic toxicity burdens were comparable between the groups (2.0, IQR 1.0–2.0 vs. 2.0, IQR 2.0–3.0; p = 0.745). The median total adverse events per patient were higher in Group A (5.0, IQR 4.0–6.0) compared to Group B (4.0, IQR 4.0–5.0), though this difference was not statistically significant (p = 0.134). Data are presented as median (IQR), and between-group comparisons were evaluated using the Mann–Whitney test.

The median hematologic adverse events per patient were significantly higher in Group A, 3.0 (IQR 2.0-3.0), compared to Group B, 2.0 (IQR 1.0-2.0) (p = 0.029). Non-hematologic adverse events were comparable between groups, with Group A reporting 2.0 (IQR 1.0-2.0) and Group B reporting 2.0 (IQR 2.0-3.0) (p = 0.745). The average number of adverse events per patient was higher in Group A, with 5.0 (IQR 4.0-6.0), compared to 4.0 (IQR 4.0-5.0) in Group B. However, this difference was not statistically significant (p = 0.134) (Figure 2).

Dose reductions and treatment delays

No dose reductions were necessary in either treatment group. Treatment delays occurred in four patients (12.1%) in the paclitaxel-carboplatin group, primarily due to neutropenia, and in three patients (9.1%) in the gemcitabine-cisplatin group, primarily due to thrombocytopenia. This difference was not statistically significant (p = 0.25). No early treatment discontinuations due to toxicity were observed.

## Discussion

The demographic and baseline clinical characteristics of the study population reveal a balanced distribution between the two treatment groups, ensuring comparability for subsequent outcome assessments.

The mean age was comparable between the two groups: 54.3 ± 8.3 years in the paclitaxel-carboplatin group (Group A) and 54.4 ± 12.3 years in the gemcitabine-cisplatin group (Group B), with no statistically significant difference (p = 0.972). This age distribution aligns with global epidemiological trends, as reported in international cancer registries, which indicate that NSCLC is most commonly diagnosed in the fifth to seventh decades of life [20]. The age homogeneity supports internal validity by reducing potential age-related bias in treatment outcomes. 

In terms of gender, male predominance was observed in both groups (63.9% in Group A and 69.7% in Group B), which is consistent with the higher incidence of NSCLC among males, as reported by Scagliotti GV. et al., who state that males predominate with a male: female ratio of 2.4:1, especially in South Asian populations, often due to higher smoking rates and occupational exposures [14, 20]. The lack of a significant sex difference (p = 0.794) suggests demographic equivalence between groups. A larger proportion of patients in both groups reported a history of smoking, with slightly more in Group B (Group A: 72.7% vs. Group B: 84.8%), indicating that tobacco use remains the primary etiological factor for NSCLC, particularly for squamous cell carcinoma [21].

Baseline performance status, a key prognostic factor in NSCLC management, was similar across groups, with an ECOG score of 0-1 (p = 0.774). This study presents evidence on the feasibility and toxicity of platinum-based chemotherapy regimens for patients with poor performance status (ECOG 2). The findings indicate that these treatments are feasible and have acceptable toxicity levels; however, survival outcomes are still inferior compared to those of patients with better performance status [15]. In terms of histology, adenocarcinoma became the most common histologic subtype, present in about two-thirds of both groups (63.6% of Group A and 69.7% of Group B), while squamous cell carcinoma made up around one-third, with a similar distribution between the groups (p = 0.794). This pattern reflects the global shift in NSCLC histopathology, where adenocarcinoma has surpassed squamous cell carcinoma as the most frequent subtype, probably due to changes in smoking habits and environmental factors [22]. The histologic balance across treatment groups further indicates that baseline disease characteristics are comparable.

Among the 66 patients assessed for treatment response, no complete responses (CR) were observed in either the paclitaxel-carboplatin (Group A) or gemcitabine-cisplatin (Group B) arms. However, more than half of the patients in each group achieved a partial response (PR), with 54.6% in each group, reflecting similar antitumor activity between the two platinum-based doublet regimens. The disease control rate (DCR) was also comparable (Group A: 78.8% vs. Group B: 75.8%) in both groups (p = 0.716). These findings are consistent with previous randomized trials and meta-analyses showing comparable response rates between these regimens when used as first-line therapy for advanced non-small cell lung cancer (NSCLC) [7]. The absence of CRs may reflect the advanced disease stage (Stage IV) of the cohort, in which systemic chemotherapy primarily serves a palliative intent to prolong survival and control symptoms rather than to achieve complete eradication [23]. Consistent overall response rates (30-32%) have been reported by Scagliotti et al. in Phase III trials comparing gemcitabine-cisplatin and cisplatin-pemetrexed; although detailed disease control (CR + PR + SD) data were not reported, stabilized disease likely contributed to comparable outcomes [20].

Short-term survival outcomes indicated modest advantages for the gemcitabine-cisplatin group at both six- and nine-month post-treatment, though the differences were not statistically significant. These findings are consistent with prior studies reporting median overall survival ranging from eight to 11 months for patients with advanced NSCLC treated with platinum-doublet regimens [24, 25]. The gradual decline in survival at nine months across both groups highlights the aggressive nature of Stage IV NSCLC and underscores the limited long-term benefit of cytotoxic chemotherapy alone, despite initial tumor response. Recent evidence suggests that a combination with immune checkpoint inhibitors or targeted agents may offer better survival outcomes in appropriately selected populations [24, 25]. However, in low- and middle-income countries (LMICs) like Bangladesh, limited access to these advanced therapies often necessitates reliance on conventional platinum-based regimens, reinforcing the clinical relevance of such comparative studies.

Regarding toxicity, distinct hematologic toxicity profiles were observed in Grades 3-4 between the two platinum-based doublet regimens commonly used in advanced NSCLC treatment. Leukopenia (75.8%) and neutropenia (78.8%) occurred significantly more often in the paclitaxel-carboplatin group. Conversely, thrombocytopenia (63.6%) was more common in the gemcitabine-cisplatin group. Gemcitabine has been shown to have a myelosuppressive effect on megakaryocytic precursors, contributing to dose-limiting thrombocytopenia, especially when administered on a Day 1 and Day 8 schedule. This aligns with the known myelosuppressive profiles of paclitaxel, which frequently causes neutropenia as a dose-limiting toxicity [24, 25].

Prolonged exposure to paclitaxel and its combination with carboplatin is linked to cumulative bone marrow suppression, especially after multiple cycles. Cisplatin can also contribute to cumulative myelosuppression, although to a lesser extent compared to carboplatin [26]. The hematologic toxicity patterns emphasize the importance of individualized toxicity monitoring and supportive care in clinical practice. These findings also contribute to the growing evidence base supporting regimen selection based not only on efficacy but also tolerability and toxicity burden, particularly in resource-constrained settings.

Non-hematologic toxicities such as nausea, vomiting, and mild hepatic and renal dysfunction were observed in both groups but did not differ significantly. These adverse events are well-documented class effects of platinum agents and may be effectively managed with prophylactic antiemetics and hydration [27]. Nausea was reported in a high proportion of patients in both groups, particularly in the paclitaxel-carboplatin arm, where rates declined from 87.9% in Cycle 1 to 63.6% in Cycle 6. The gemcitabine-cisplatin group showed slightly lower but still considerable rates, ranging from 72.8% to 54.5%. Vomiting was less frequent across both regimens, ranging from 15.2% to 27.3% across treatment cycles. Although specific cycle-by-cycle percentages are not available in a single report, the Phase III trial in *Annals of Oncology *confirms that nausea and vomiting are significantly more frequent with paclitaxel plus cisplatin than with paclitaxel plus carboplatin. This aligns with the known emetogenic potential of platinum-based regimens, particularly when combined with taxanes [28]. 

Mild elevations in SGPT (ranging from 6.1% to 12.1%) and serum creatinine levels up to 12.1% were observed in both treatment groups, with no statistically significant differences between the groups. These findings are consistent with phase III NSCLC trials of gemcitabine-cisplatin, in which Grade 2 ALT elevations occurred in approximately 10% of patients and Grade 2 creatinine elevations in around 9%, while higher-grade events were uncommon [29]. Transient, mild-to-moderate hepatic and renal laboratory abnormalities are expected with gemcitabine-cisplatin, and clinically significant liver injury or nephrotoxicity remains rare [30].

In addition to treatment-related toxicities, we conducted an exploratory analysis stratifying patients by age groups (<60 and ≥60 years) to evaluate potential differences in survival and hematologic toxicity. We found that younger patients (<60 years) appeared to have slightly better short-term survival and lower rates of severe hematologic toxicity compared to older patients. However, these trends were not statistically significant, likely due to the small sample size and limited power of this study. These findings should therefore be interpreted cautiously and explored further in larger, multicenter studies.

In this study, neither treatment regimen required dose reductions, and treatment delays were infrequent and comparable between groups (Group A: 12.1% vs Group B: 9.1%; p=0.25). Neutropenia was more prevalent in the paclitaxel-carboplatin arm, while thrombocytopenia occurred more frequently in the gemcitabine-cisplatin arm, consistent with established toxicity profiles [28]. The absence of early discontinuations suggests that both regimens were well tolerated in this setting, despite the differences in hematologic adverse event patterns.

Limitations

This study has certain limitations, including its single-center setting, small sample size, and non-randomized allocation, which may limit the generalizability of the results to broader populations and introduce selection bias due to treatment allocation based on physician judgment and patient preference, potentially limiting internal validity. However, the prospective design enhanced the reliability of data collection and follow-up, providing valuable real-world insights into treatment outcomes for patients with Stage IV NSCLC in a resource-limited setting. Future multicenter, long-term randomized studies are needed to validate these findings.

## Conclusions

This study has certain limitations, including its single-center setting, small sample size, and non-randomized allocation, which may limit the generalizability of the results to broader populations and introduce selection bias due to treatment allocation based on physician judgment and patient preference, potentially limiting internal validity. However, the prospective design enhanced the reliability of data collection and follow-up, providing valuable real-world insights into treatment outcomes for patients with Stage IV NSCLC in a resource-limited setting.
